# How to Early Identify and Prevent the SARS-CoV-2 Infection in Children for Families?

**DOI:** 10.3389/fped.2020.00542

**Published:** 2020-09-04

**Authors:** Meng-Yuan Qiao, Na Chen, Xian Zou, Dan-Hua Mao, Hong-Tao Cui, Wei-Bin Li, Jing-Kun Miao, Qi-Xiong Chen

**Affiliations:** ^1^Department of Neonates, Children's Hospital of Chongqing Medical University, Chongqing, China; ^2^China International Science and Technology Cooperation Base of Child Development and Critical Disorders, Chongqing, China; ^3^Ministry of Education Key Laboratory of Child Development and Disorders, Chongqing, China; ^4^National Clinical Research Center for Child Health and Disorders, Chongqing, China; ^5^Chongqing Key Laboratory of Pediatrics, Chongqing, China; ^6^Chongqing Traditional Chinese Medicine Hospital, Chongqing, China; ^7^Chongqing Maternal and Child Health Care Hospital, Chongqing, China

**Keywords:** COVID-19, novel coronavirus, pediatrics, SARS-CoV-2, identification

## Abstract

**Importance:** COVID-19 has become a worldwide pandemic. Many countries have reported cases of infection in children and newborns, and there is a trend of significantly increasing infections among these populations. Therefore, it is important to provide advice and guidance for the prevention and control of COVID-19 in children.

**Observations:** Children are as susceptible to SARS-CoV-2 infection as adults. The manifestations in children are atypical, and children are much less likely to have critical cases. If children are infected, they may play an important role in the spread of SARS-CoV-2 because their symptoms are less obvious and less likely to be detected. To prevent COVID-19 from spreading among children, efforts to prevent, and control the infection should be increased by controlling the source of infection, blocking the route of transmission and protecting the susceptible population.

**Conclusions and Relevance:** The early identification of the COVID-19 in children and the protection of families are important measures to prevent the continued spread of SARS-CoV-2.

## Introduction

Since December 2019, there has been an epidemic of novel coronavirus infections in China (1), and it has quickly spread across China and to 211 other countries. By April 27, 2020, a total of 2,878,196 cases have been confirmed worldwide. A total of 84,341 cases have been confirmed in China; among these individuals, 4,643 patients have died, with a fatality rate of 5.51%, and there are more suspected cases in children. Among other countries, the United States of America (931,698 cases), Spain (207,634 cases), Italy (197,675 cases), Germany (155,193 cases), France (123,279 cases), and the United Kingdom (152,844 cases) have the most serious outbreaks (2). The International Virus Classification Committee officially named the causative pathogen severe acute respiratory syndrome coronavirus 2 (SARS-CoV-2), and the WHO also declared that the disease caused by SARS-CoV-2 was officially named coronavirus disease 2019 (COVID-19) (3). As the COVID-19 outbreak is regarded as a global epidemic, global prevention, and control work still face huge challenges. With the progression of the epidemic and the development of etiological detection, there has been a significant increase in the number of reported cases of infection in children, even in infants and newborns. Here, we summarize the epidemiological characteristics, clinical manifestations, diagnosis, and prevention of COVID-19 in children and examine differences between infection in children and adults to provide a reference for pediatric clinicians and to aid in the prevention and control of infections among families worldwide.

## Search Strategy and Selection Criteria

References for this review were identified through searches of PubMed, Wanfang database, and CNKI (China National Knowledge Infrastructure) and included articles published from January 1971 to April 2020 that were identified with the terms “SARS-COV-2,” “COVID-19,” “novel coronavirus,” and “children.” Articles retrieved from these searches and relevant references cited in those articles were reviewed. Articles published in English and Chinese were included.

## Epidemiological Characteristics

### Source of Infection

At present, the source of infection is mainly SARS-CoV-2 infected patients, but asymptomatic infection and other people who discharge the virus (in the incubation period and recovery period) are also important (4).

### Route of Transmission

The main route of transmission is respiratory droplets, which are transmitted as droplets when patients cough, speak loudly, and sneeze. Contact transmission is another important route when contaminated hands (from contact with patient secretions, feces, and other contaminants) touch the mouth, nose, and eye mucosa.

There is a possibility of aerosol transmission with long-term exposure to high concentration aerosols in a relatively closed environment. As viruses can be isolated from feces and urine, attention should be paid to aerosol or contact transmission caused by feces and urine pollution (4). Some studies have proposed potential evidence of continuous fecal detoxification, but there is no evidence of a virus with strong replication from fecal swabs. Thus, the possibility of fecal-oral transmission remains unclear (5).

At the same time, a study found that IgM antibodies to SARS-CoV-2 increased in a newborn whose mother had COVID-19, which suggests that we need to be aware of the possibility of vertical transmission (6). However, for newborns and breastfeeding infants, whether it can be transmitted through mother-to-child vertical transmission or through breast milk transmission needs to be further studied (7, 8).

### Susceptible Populations and Epidemic Characteristics

Children are susceptible to novel coronavirus throughout the year. Younger children are vulnerable to infection and may experience more severe outcomes (9, 10). At present, domestic case data show that children under the age of 18 account for 2.4% of all reported cases. A recent review of 72,314 cases by the Chinese Center for Disease Control and Prevention showed that <1% of cases in China occurred in children under the age of 10 (11). Two children who tested positive for COVID-19 have died in China. A 14-year-old boy from Hubei Province who died was among the 2,135 children with COVID-19 (9). A 10-month-old child with intussusception died 4 weeks after admission because of multiple organ failure (12).

The existing epidemiological data show that 26.8% of patients had a history of exposure to the epidemic center, and 71.2% of patients had a history of a family cluster of infection (13). Four cases of neonatal infection have been reported in China, among children ranging in age from 30 h to 17 days. A 30-h-old male child born by cesarean section to a woman diagnosed with COVID-19, was diagnosed after a positive nucleic acid test. A 17-day-old male child was diagnosed after the diagnosis of a home nursing staff member (the first infection) and his mother. A 5-day-old male child was diagnosed with COVID-19 after developing fever, and his mother was also diagnosed. A 5-day-old female, delivered by a mother with COVID-19, was diagnosed after nucleic acid detection, although she had no symptoms (14).

## The Clinical Characteristics of SARS-CoV-2 Infection are Different in Children and Adults

### Mild Clinical Manifestations and few Severe Cases

Compared with adult patients, pediatric patients have characteristics that include a lower incidence, milder clinical manifestations, shorter course of disease, fewer severe cases, and a better prognosis (13), which is similar to SARS infections in 2003. However, the possibility of asymptomatic infection or missed diagnosis in children cannot be ruled out. Younger children are susceptible to the disease and may experience more serious outcomes. Therefore, close monitoring is important, especially for children with underlying diseases, as is striving for early identification and timely treatment (10).

According to current reported data on children reported at present, most of the clinical symptoms are relatively milder in pediatric patients than in adult patients, and children may even have no symptoms of fever or pneumonia. A study of the epidemiological characteristics of 2,135 pediatric patients under 18 found that 94 (4.4%), 1,088 (51.0%), and 826 (38.7%) children were diagnosed with asymptomatic cases, mild cases, or moderate cases, respectively, which suggests that the clinical manifestations of COVID-19 in children were generally less severe than those in adults. At the same time, the data show that young children, especially newborns, are very vulnerable to infection (9). The clinical manifestations of COVID-19 may be less severe in children than in adults because children are well-protected at home and have fewer opportunities for exposure to patients infected with COVID-19. ACE2 has been identified as a functional host receptor for SARS-CoV-2 (15). It has been speculated that the function and maturity of ACE2 in children are lower than those in adults, which makes them less sensitive to COVID-19 (16). In addition, the immune systems of children are immature and may respond to pathogens less strongly than those of adults. Furthermore, children often suffer from respiratory infections in the winter and may have higher levels of viral antibodies than adults.

Fever and cough were more common in 134 confirmed pediatric infections in an epidemiological survey of China. Among the 117 patients with temperature records, 89 patients had fever, and 28 patients did not have fever (13). Date from a study of 171 pediatric patients with COVID-19 showed that 71 patients had fever and 100 patients had no fever (<37.5°C). Among patients with fever, 16 had high fever(>39.0°C), 39 had moderate fever (38.1–39.0°C), and 16 had low fever (37.5–38.0°C) (12). This suggests that fever is less obvious in children than adults, and the ratio of mild fever to non-fever cases seems to be higher in children than adults. Therefore, it is easy to miss a diagnosis if we are simply concerned with fever.

Some infected children also present with fatigue, myalgia, stuffy nose, runny nose, sneezing, sore throat, headache, dizziness, nausea, vomiting, abdominal pain, and diarrhea. Recently, it was reported that a 45-day-old infant only presented with frequent vomiting in the early stage and gradually progressed to a severe case (13). Newborns, especially premature infants, are more likely to present with hidden and nonspecific symptoms, such as body temperature instability, low activity, improper feeding, or shortness of breath (14, 17). It is important to pay attention to symptoms other than fever and cough in suspected cases with an epidemiological history.

### Family Clusters May Be the Most Important Clue for the Diagnosis of Pediatric Cases

The characteristics of disease cluster are particularly obvious in pediatric cases of this epidemic. In the nine infants with SARS-CoV-2 infections reported in JAMA, the common feature was that there were cases of infection cases in all the family members of the children (18). In a study of 10 children with COVID-19, four patients had a clear history of contact with diagnosed patients, seven patients came from families with infection clusters, and seven patients had a history of travel to the epidemic area of Hubei Province 2 weeks before the onset of the disease (5). Another study reported that the route of transmission in pediatric patients was either through close contact with family members carrying COVID-19 (32 [89%]), through a history of exposure to epidemic areas such as Wuhan Province (12 [33%]), or both (8 [22%]) (19). At the same time, by analyzing the epidemiological data of 285 confirmed pediatric cases reported in China until February 7, 2020, a study found that 26.8% (69/257) had a history of exposure to an epidemic area, and 71.2% (183/257) had a clear history of family infection cluster (13).

Among the four newborns infected with COVID-19, their mothers all had SARS-CoV-2 infections. Three mothers were diagnosed and showed symptoms before delivery, and 1 was diagnosed and showed symptoms after delivery. Therefore, it is necessary to inquire whether family members have an epidemiological history and clinical manifestations of COVID-19 for children with suspected infection, and family members can be examined with laboratory and imaging examinations for COVID-19 if necessary.

### Decreased White Blood Cell Count, Lymphocyte Count and Elevated C-Reactive Protein Are Rare and Cannot Be Clinically Treated According to the Criteria for Adult Cases

In adult cases of infection, the peripheral blood lymphocyte count significantly or progressively decreased in the early stage of the disease (20), and the levels of both CD4^+^ and CD8^+^ lymphocytes decreased. However, the white blood cell count and lymphocyte count were normal in most of pediatric cases, and there was no lymphocyte decrease. This phenomenon may be related to the incomplete development of innate immunity, which may lead to a low level of adaptive immune response in children, which may explain the clinical characteristics of mild disease in children. Through the analysis of the laboratory examinations of 36 children with COVID-19 infections, it was found that only 11 (31%) had decreased lymphocytes, 7 (19%) had leukopenia, and increased C-reactive protein (2 [5%]) was rare (19). Through routine blood tests of 134 children, it was found that only 2 patients had a slight decrease in white blood cell counts, and only 1 patient had a slight decrease in lymphocyte counts (9 years old, 0.78 × 10^9^/L). It has been suggested that children with infections are less likely to have a cytokine storm caused by an excessive immune response, so leukopenia and lymphocyte decreases are relatively rare. Similarly, in the detection of C-reactive protein, only 3 of 134 patients had transient increases in C-reactive protein (23, 27, and 47 mg/L) (13).

## Clinical Classifications

According to the clinical manifestations, children with COVID-19 were classified into five groups: asymptomatic infection and mild, moderate, severe, and critical cases (9, 13).

### Asymptomatic Infection

Children have no positive clinical symptoms or signs, and the chest imaging examination is normal, while the result of the 2019-nCoV nucleic acid test is positive.

### Mild Cases

Children have symptoms of acute upper respiratory infection, including fever, fatigue, cough, sore throat, myalgia, runny nose, and sneezing. The physical examination shows that the pharynx is congested and that there is no abnormal auscultation. Some cases may not have fever or may have only digestive symptoms such as nausea, abdominal pain, vomiting, and diarrhea.

### Moderate Cases

Children have pneumonia, and the clinical manifestations often include frequent fever and cough (mainly dry cough, followed by productive cough); some may have wheezing, but there is no obvious hypoxemia, such as shortness of breath. Phlegm, wet rales or dry rales can be heard in the lungs. In some cases, there may be no clinical signs or symptoms, but chest CT shows subclinical lung lesions.

### Severe Cases

Children have early respiratory symptoms such as fever and cough, which may be accompanied by gastrointestinal symptoms such as diarrhea. The disease usually progresses after approximately 1 week, and dyspnea occurs and is accompanied by central cyanosis. Oxygen saturation (SpO_2_) is <92%, with other hypoxic manifestations.

### Critical Cases

Children can quickly develop acute respiratory distress syndrome (ARDS) or respiratory failure and may also have encephalopathy, shock, myocardial injury or heart failure, acute kidney injury, and coagulation dysfunction. Organ dysfunction can be life-threatening.

## Clinical Early Warning Index of Severe and Critical Infections (4, 13)

### High-Risk Group of Children (21, 22)

According to the experience of diagnosing and treating community-acquired pneumonia in children and the current clinical treatment practice for confirmed cases, the high-risk groups that may develop severe or critically ill cases include the following:

(1) Patients with underlying diseases such as congenital cardiopulmonary airway disease, chronic heart and kidney disease, malnutrition, cancer, diabetes, immune deficiency, hypothyroidism, genetic metabolic diseases, etc. (23, 24).
(2) Patients with long-term use of immunosuppressants (25).

### Early Warning Index

Children who meet any of the following criteria may develop a severe or critically ill case:

(1) Increased respiratory rate, defined as RR > 50/min (2–12 months), RR > 40/min (1–5 years), and RR > 30/min (>5 years), excluding the effects of fever and crying;
(2) Persistent high fever for 3–5 days, a disease course of more than 1 week, no improvement of symptoms and signs or progression.
(3) Poor mental reactions, drowsiness, etc.
(4) Significant and/or progressive decreases in peripheral blood lymphocytes;
(5) Progressive increases in enzymatic indexes, such as myocardial enzymes, liver enzymes, lactate dehydrogenase, etc.;
(6) Unexplained metabolic acidosis.
(7) Significantly increased D-dimer, IL-6, IL-10, and ferritin;
(8) Oxygen saturation (SpO_2_) below 95% at rest;
(9) Extrapulmonary complications; and
(10) Mixed infection with other viruses and/or bacteria.

## Diagnostic Criteria (13)

The diagnostic criteria are mainly based on the analysis of the epidemiology and clinical manifestations.

### Epidemiology

(1) Travel or residence history in Wuhan and its surrounding areas or other epidemic areas with case reports within 14 days before the onset of the disease.
(2) Contact with patients with fever or respiratory symptoms from Wuhan and its surrounding areas, or from a community with reported cases within 14 days before the onset of disease.
(3) Close contact with confirmed (nucleic acid test positive) or suspected COVID-19 cases within 14 days before the onset of disease.
(4) Clustered onset (there were 2 or more cases of fever and/or respiratory symptoms in small groups, such as families, offices, school classes, etc., within 2 weeks). There were other patients with fever or respiratory symptoms besides the child, including suspected or confirmed cases of COVID-19 or newborns with mothers who are suspected to have or diagnosed with COVID-19.

### Epidemiological Grading of Children

(1) High Risk: Close contact with suspected or confirmed cases of SARS-CoV-2 infection within 14 days before the onset of disease.
(2) Moderate risk: Cluster onset of COVID-19 occurred in the place of residence or community.
(3) Low risk: There was no cluster onset in the community, and children stayed away from the epidemic area.

### Clinical Manifestations

(1) Fever, fatigue, dry cough, and some children may not have fever or low-grade fever.
(2) Imaging features of pneumonia mentioned above.
(3) The total leukocyte count is normal or decreased or the lymphocyte count decreases in the early stage of the disease.
(4) There are no clinical manifestations that can be completely explained by other etiologies.

### Definition of Suspected and Confirmed Cases in Children

#### Suspected Case

Newborns born to women with confirmed COVID-19 are suspected cases. High-risk children who meet any two of the following criteria are suspected cases. Cases at medium or low risk who meet any two of the following criteria are suspected cases after excluding influenza and other common respiratory pathogens.

1) Fever, respiratory symptoms, shortness of breath, or decreased pulse oxygen saturation, or gastrointestinal manifestations such as nausea, vomiting, abdominal discomfort, or diarrhea.
2) Laboratory examinations: the total leukocyte count is normal or decreased, lymphocyte count is decreased, and C-reactive protein is normal or slightly increased.
3) Pulmonary imaging shows signs of pneumonia caused by SARS-CoV-2.

#### Confirmed Cases

The suspected case has one of the following etiological or serological signs at the same time:

1) SARS-CoV-2 nucleic acid is positive by real-time fluorescence RT-PCR;
2) Virus gene sequencing is highly homologous to the novel coronavirus; and
3) Serum novel coronavirus-specific IgM and IgG are positive, and serum novel coronavirus-specific IgG changes from negative to positive or increases by at least 4-fold in the acute stage.

It is worth noting that even if the pharynx swab real-time fluorescence PCR detection of SARS-CoV-2 nucleic acid is negative, suspected cases should not be easily excluded; lower respiratory tract samples should be tested or repeated nucleic acid detection in upper respiratory tract samples should be performed.

## Prevention and Control Measures

The epidemic is still the growth stage at present. Combined with the current cases in children, there will not be many cases of severe infection in children, but there is still virus excretion in children after achieving a clinical cure. Therefore, efforts should be increased to prevent and control pediatric infection in three areas to prevent the COVID-19 epidemic from spreading among children: controlling the source of infection, blocking the route of transmission and protecting the susceptible population (26, 27).

### Management of Infection Sources

The most important measure for children diagnosed with COVID-19 is quarantine. Children with suspected and confirmed COVID-19 should be quarantined in the hospital to observe changes in their condition and to receive treatment in a hospital under the guidance of medical staff. For newborns born to mothers with confirmed asymptomatic or paucisymptomatic infection at delivery, the Union of European Neonatal and Perinatal Societies (UENPS) suggests that direct breastfeeding under strict measures of infection control is advisable. However, if a mother is too sick to care for the newborn, the neonate should be cared for away from the mother and fed fresh expressed breast milk (with no need to pasteurize it) (28). According to current research findings, there is no strong evidence about whether COVID-19 infection might occur via transplacental transmission. In a study on nine women with COVID-19, SARS-CoV-2 was not found in the amniotic fluid, in cord blood, or in breast milk, which means that neonatal COVID-19 might be the result of transmission from the mother via a respiratory route (8). Therefore, we recommend breastfeeding given its benefits for the health of mothers, children, and society.

### Management of Transmission Routes

Due to children's low immunity, low compliance, low masking, and other reasons, personal protection should be increased with the following methods.

(1) Although the use of face masks to prevent transmission is still being discussed, we already know that the use of protective masks in medical institutions plays an important role in reducing respiratory tract infections among medical staff. A study (29) conducted in 2006–2007 showed for with a virus with an incubation period of 1 or 2 days, the use of an N95 or surgical mask significantly reduced the risk for ILI (influenza-like illness) infection in households. Although the subsequent experiment was different due to decreased adherence for wearing masks, the results still indicated that the potential efficacy of correctly wearing masks may be expected during an influenza pandemic or other infection. Therefore, for viruses such as SARS-CoV-2, which is transmitted through the respiratory tract or aerosols and has a long incubation period, it is clear that people should wear masks and maintain social distance when they go out to reduce the spread of the virus. Thus, we recommend that children correctly wear masks under the guidance of parents, preferably using special masks for children, and parents should pay attention to the breathing of children. It is not recommended that children under 1 year wear masks, and N95 masks are not recommended for children under 7 years of age to prevent asphyxiation.
(2) Public health measures played an important role in controlling the SARS epidemic in 2003. 2019-nCoV is thought to be transmitted mainly by respiratory droplets, and its incubation time and generation time are similar to SARS coronavirus (SARS-CoV). Thus, in the absence of antivirals and vaccines, we may need to rely on classic public health measures to curb the epidemic of this respiratory disease (30). Social or physical distancing, including maintaining distance from other people and closing schools, is designed to effectively slow the spread of disease between children by minimizing close contact between individuals (31). Some people argued that some of these methods may infringe upon citizens' civil liberties, and some of these measures are referred to as “draconian.” However, we should not only consider individual rights, but also take the rights of those who are not infected but are at risk of infection into account. The Centers for Disease Control and Prevention in the United States have established clinical guidance for schools to slow the transmission of SARS-CoV-2. These measures include school dismissals, event cancellations, and other social distancing measures (32). And an observational study showed that nonpharmaceutical interventions (including school closures, isolation, distancing, and behavior change in populations) were effective in reducing the transmission of COVID-19 in Hong Kong (33).
(3) Teach children to wash their hands correctly and frequently (washing hands with an alcoholic-based hand sanitizer or using soap and water for 20 s). The room should be frequently ventilated, and at the same time, additional clothing should be worn to avoid catching cold. Properly disinfect the home and create a clean environment. Items that are used daily should be regularly cleaned with household cleaners.
(4) Try to take private cars and other means of transport when going out, reducing the gathering of individuals.

### Management of Susceptible People

The population is generally susceptible, among whom children and newborns may be more susceptible because of low immunity. The protection of susceptible people should be achieved with the following methods (34).

(1) Parents should ensure children's hygiene, frequently wash hands, improve family hygiene and disinfection, prevent children from catching cold, and prevent cross-infection.
(2) Ensure that children received adequate nutrition and a balanced diet; we should avoid eating raw food, wild animals, fresh food, etc.
(3) During the epidemic, it is necessary to avoid going out as much as possible. Reasonable adjustments should be made to childcare and vaccination clinics, and the collective activities of childcare institutions or schools need to be changed to reduce crowded gatherings. Children with special health care needs, such as asthma and diabetes, might need to avoid crowds more than other children.
(4) Children should also cultivate good behavior habits, work and rest regularly, and young children should make reasonable arrangements for their study and life at home (35).
(5) Children may have abnormal emotions such as fear, irritability, and anxiety during the epidemic. Appropriate psychological support in the context of a virus outbreak should also include children.
(6) If children get sick, please keep them at home and ask the doctor for help online. Do not go to an emergency department unless you are having an emergency.

## Conclusion

Children are as susceptible to SARS-CoV-2 infection as adults, but they are much less likely to develop critical cases. If children are infected, they may play an important role in the spread of SARS-CoV-2 because their symptoms are less obvious and less likely to be detected. Thus, the early identification of COVID-19 infection in children and the protection of families are important measures for preventing the continued spread of SARS-CoV-2.

## Author Contributions

M-YQ and NC conceptualized and designed the study and drafted the initial manuscript. XZ, D-HM, and H-TC wrote and revised the manuscript. J-KM and W-BL helped with selection of research and the construction of article. Q-XC designed the study, reviewed, and revised the manuscript. All authors approved the final manuscript as submitted and agree to be accountable for all aspects of the work.

## Conflict of Interest

The authors declare that the research was conducted in the absence of any commercial or financial relationships that could be construed as a potential conflict of interest.
